# Relationship Between an *Interleukin 6* SNP and Relapse After Allogeneic Bone Marrow Transplantation

**DOI:** 10.3390/jcm14020476

**Published:** 2025-01-13

**Authors:** Hidekazu Takahashi, Natsu Yamaguchi, Naoko Okayama, Mitsuaki Nishioka, M. H. Mahbub, Ryosuke Hase, Yutaka Suehiro, Takahiro Yamasaki, Satoshi Takahashi, Arinobu Tojo, Tsuyoshi Tanabe

**Affiliations:** 1 Department of Public Health and Preventive Medicine, Yamaguchi University Graduate School of Medicine, Ube 755-8505, Japan; hidetakaha4@gmail.com (H.T.); natsu@yamaguchi-u.ac.jp (N.Y.); hossain@yamaguchi-u.ac.jp (M.H.M.); hase@yamaguchi-u.ac.jp (R.H.); 2 Division of Laboratory, Yamaguchi University Hospital, Ube 755-8505, Japan; nokayama@yamaguchi-u.ac.jp (N.O.); mnishi@yamaguchi-u.ac.jp (M.N.); ysuehiro@yamaguchi-u.ac.jp (Y.S.); t.yama@yamaguchi-u.ac.jp (T.Y.); 3 Division of Medical Genetics, Yamaguchi University Hospital, Ube 755-8505, Japan; 4 Department of Oncology and Laboratory Medicine, Yamaguchi University Graduate School of Medicine, Ube 755-8505, Japan; 5 Division of Clinical Precision Research Platform, The Institute of Medical Science, The University of Tokyo, Tokyo 113-8654, Japan; radius@ims.u-tokyo.ac.jp; 6 Tokyo Medical and Dental University, Tokyo 113-8510, Japan; tojo.adm@tmd.ac.jp

**Keywords:** hematopoietic stem cell transplantation, bone marrow transplantation, single nucleotide polymorphism, association study, cytokine, interleukin

## Abstract

**Background/Objectives:** Unrelated bone marrow transplantation (BMT) is a curative treatment for hematological malignancies. While HLA mismatch is a recognized risk factor in unrelated BMT, the significance of non-HLA single nucleotide polymorphisms (SNPs) remains uncertain. Cytokines play key roles in several aspects of unrelated BMT. Although the relationship between cytokine gene SNPs and BMT outcomes has been examined, the findings obtained have been inconsistent; therefore, further investigations in additional cohorts are warranted. **Methods:** Four SNPs in the *IL2*, *IL6*, *IFN-gamma*, and *TGF-beta1* genes were retrospectively genotyped in 822 malignant patients and their corresponding donors who received unrelated BMT through the Japan Marrow Donor Program with compatibility at minimum HLA-A, -B, and -DRB1. The relationships between these SNP genotypes and BMT outcomes were statistically analyzed. **Results:** The donor *interleukin-6* (*IL6*) SNP, rs1800796, also known as -572G>C and -634C/G, was associated with the relapse of the original disease in both univariable and multivariable regression analyses (minimum *p*-value = 0.0013), and the cumulative incidence curve analysis identified CC as a risk genotype (*p*-value = 0.0012). None of these SNPs correlated with overall survival. **Conclusions:** The donor *IL6* SNP, rs1800796, may serve as a useful predictor of tumor relapses if validated.

## 1. Introduction

Hematopoietic stem cell transplantation (HSCT) offers a curative approach for blood-related diseases [1,2]. Allogeneic HSCT encompasses various forms, like bone marrow transplantation (BMT) and cord blood transplantation, based on the source of the hematopoietic stem cells used. While post-allogeneic HSCT patient outcomes have seen advancements in recent years, further advances are desired [3].

In allogeneic HSCT, HLA matching between a recipient and donor is generally very important [4,5,6]. However, even if the HLA is fully matched, the five-year survival rate after transplantation does not reach close to 100%, and relapse of tumors and severe post-transplant complications, such as graft-versus-host disease (GVHD), continue to occur. Therefore, single nucleotide polymorphisms (SNPs) outside the HLA locus may also be important for HSCT [7]. SNPs in genes in the immune system may affect allogeneic HSCT outcomes due to changes in immunity. The identification of these SNPs is useful not only for obtaining a more detailed understanding of allogeneic HSCT biology but also potentially for predicting outcomes and selecting donors.

However, association studies on non-HLA SNPs in humans have generally not yielded consistent findings [7,8,9,10,11,12,13]. Although the exact reason(s) are unclear, this may be due in part to the presence of more potentially confounding factors, including a recipient’s underlying disease, donor characteristics, treatment procedures, and HLA matching in allogeneic HSCT, than in other diseases. Differences in genetic ancestry may affect the findings of human allogeneic HSCT studies. Furthermore, SNP–SNP interactions and SNP–covariate interactions may play roles. In any case, more association studies in diverse settings and populations are needed.

Our previous studies explored the associations between unrelated BMT outcomes and SNPs within genes of innate immune pathway, including *NOD2* and *NLRP3*, immune checkpoint genes, such as *PD1* and *BTLA*, and the methylase genes, *DNMT1* and *EZH2* [14,15,16]. However, we have not yet identified any SNP that is associated with unrelated BMT outcomes as a single independent variable, except SNP-covariate interactions.

Cytokines are secreted factors that play important roles in several aspects of allogeneic HSCT, including GVHD and T cell differentiation [17]. The relationship between cytokine gene SNPs and HSCT has been examined [8,18,19,20,21,22,23,24,25,26,27,28,29,30]; however, similar to SNPs in other genes, the findings obtained have been inconsistent and, thus, further investigations in additional cohorts are warranted. In this study, we investigated the relationships between SNPs in the *IL2*, *IL6*, *IFN-gamma*, and *TGF-beta1* genes, which do not apparently function in the same pathways as the aforementioned genes we have analyzed so far, and the outcomes of unrelated BMT matched at least at HLA-A, -B, and -DRB1, performed between May 2006 and April 2009 under the Japan Marrow Donor Program (JMDP).

## 2. Subjects, Materials, and Methods

### 2.1. Subjects

Donor and patient characteristics are shown in Supplementary Table S1 and have been described in detail [15]. Briefly, retrospective genotyping was conducted for 999 Japanese recipients and their corresponding donors undergoing unrelated BMT between May 2006 and April 2009 through the JMDP [31] and meeting the following criteria: donors aged 20 years or older; survival data availability; and matched alleles for the HLA-A, -B, and -DRB1 (matching for the HLA-C, -DPB1, and -DQB1 alleles was not obligatory). Among these, 822 cases involving patients with malignancies and no history of prior transplants, along with their respective donors, formed the core dataset (Supplementary Table S1). The Japanese Society for Transplantation and Cellular Therapy collected clinical data through the Transplant Registry Unified Management Program [32].

### 2.2. SNP Selection

We identified SNPs that either encode non-synonymous amino acid substitutions or are known to influence gene expression, with a minor allele frequency (MAF) greater than 0.1 in 104 Japanese residents from Tokyo (JPT104) in the 1000 Genomes Project [33] (Supplementary Table S2).

The *IL2* SNP, rs2069762, also known as -330, is located in the promoter region, and peripheral blood lymphocytes from healthy volunteers with the GG genotype have been shown to produce more IL2 than those with the TT and GT genotypes due to an early and sustained enhancement [34].

The *IL6* SNP, rs1800796, also known as -572G>C and -634C/G, is located in the promoter region, and Japanese individuals carrying the -634G allele have been shown to secrete more IL-6 from peripheral blood mononuclear cells when stimulated [35].

The *IFNG* SNP, rs2069705, (known as -1616T/C), resides in the 5′ untranslated region (UTR), with the A allele correlating to reduced IFN-γ levels [36].

The *TGFB* SNP, rs1800469, also known as -509C>T and -1347C>T, is located in a negative regulatory region of the promoter, and its A(T) allele has been reported to increase RNA transcription by preventing AP1 binding [37].

### 2.3. SNP Genotyping

Genomic DNA was extracted and purified from 200 μL of peripheral blood collected from each donor and recipient using the QIAamp DNA Blood Mini Kit (Qiagen, Germantown, MD, USA). The purified DNA was then amplified using the Illustra GenomiPhi HY Kit (GE Healthcare, Tokyo, Japan), following the previously described protocol [15]. SNP genotyping was conducted as previously described [15] with TaqMan SNP Genotyping Assays (Applied Biosystems, Carlsbad, CA, USA), the assay IDs of which are given in Supplementary Table S2. A default software threshold of 95 for quality was applied to all SNPs. Genomic DNA samples, which were not successfully genotyped with TaqMan assays, were genotyped by direct Sanger sequencing following PCR using the primers shown in Supplementary Table S2, as previously described [15]. The latter sequencing approach was also used to verify the results of the former TaqMan approach: around ten samples for each of the three genotypes, two homozygous and one heterozygous, underwent sequencing.

### 2.4. Statistical Analysis

Statistical analyses followed previously outlined methodologies [15,16]. Overall survival (OS) was assessed through the Cox proportional hazard regression, while other outcomes were evaluated using the Fine–Gray proportional sub-distribution hazard regression. In the regression analysis, *p* values were determined using the Wald test. In one type of multivariable regression, a single SNP was fixed, and the clinical variables are listed in Supplementary Table S1, excluding the underlying disease, which exhibited *p* < 0.1 without violating the proportional hazards assumption in the univariable regression and were subjected to backward variable elimination based on the Bayesian information criterion (BIC). All product interaction terms between the SNP and other retained covariates were then introduced and subjected to another round of backward elimination with fixed non-interaction terms. In all elimination steps, the covariates that not only improved the BIC of the model but also exhibited significance were retained in the model. In another type of multivariable regression, reported risk factors were fixed as covariates to ensure that the results obtained were comparable with previous findings [15,16]. The fixed covariates for the multivariable regression of non-relapse mortality (NRM) and relapse matched those applied in the multivariable regression of OS. These covariates were selected based on recipient characteristics, donor characteristics, transplantation variables, and the recipient–donor matching status, as detailed in Supplementary Table S1, and were previously described [15]. HLA mismatches were counted in the graft-versus-host direction, unless noted otherwise. All *p* values reported are unadjusted and two-tailed. In the present study, we used a significance level of 0.0025, half the threshold used in previous studies, to minimize the risk of false positives due to multiple comparisons. The significance of cumulative incidence curves was evaluated using a Gray’s test. Analyses were performed using R software (ver. 3.2), EZR software [38], and Microsoft Excel.

## 3. Results

### 3.1. Genotyping

We successfully genotyped all four SNPs derived from the *IL2*, *IL6*, *IFNG*, and *TGFB* genes in all recipients and their donors (Supplementary Table S3). As expected, comparable allele frequencies were observed among 887 first-time transplantation recipients, their donors, and 104 Japanese residents of Tokyo (JPT104) in the 1000 Genomes Project [33] (Supplementary Table S4). Since the Hardy–Weinberg equilibrium null hypothesis was not refuted for these SNPs (Supplementary Table S4), they were all incorporated into subsequent statistical analyses.

### 3.2. Grade 2–4 Acute GVHD (aGVHD)

A univariable regression analysis was conducted for the primary outcome, grade 2–4 aGVHD, focusing on these four SNPs. None of the SNPs, regardless of donor or recipient, correlated with grade 2–4 aGVHD (Supplementary Table S5). Of these SNPs, the *IL2* SNP, rs2069762, in donors showed the lowest *p*-value under the additive model (*p* = 0.018). We then conducted a multivariable regression analysis that used BIC-based covariate selection and considered interaction terms [15]. Neither SNP variables nor SNP–covariate interaction terms were correlated with grade 2–4 aGVHD. Additionally, a multivariable regression was performed, which was adjusted for reported covariates relevant to grade 2–4 aGVHD without considering interaction terms to make comparisons with previous studies possible. None of the SNPs exhibited any significant correlation with grade 2–4 aGVHD (Supplementary Table S6).

### 3.3. Grade 3–4 aGVHD

In both univariable regression and multivariable regression analyses, no significant associations were observed between any SNP and grade 3–4 aGVHD (Supplementary Tables S7 and S8). Among these SNPs, the donor *IL2* SNP, rs2069762, which exhibited the smallest *p*-value in grade 2–4 aGVHD, as mentioned above, exhibited even smaller *p*-values in univariable and multivariable regressions (*p*~0.0035 and 0.0042, respectively).

### 3.4. Relapse

**Figure 1:**
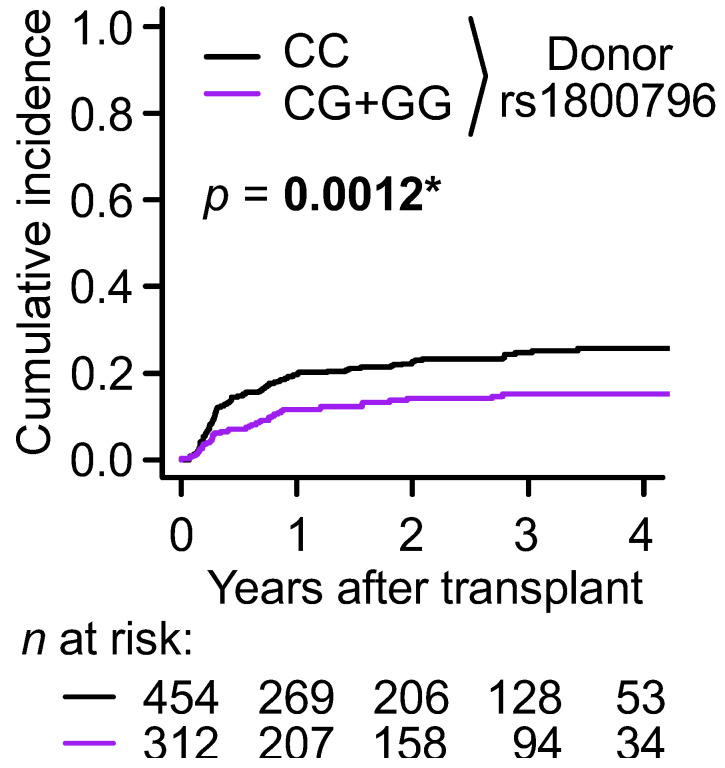
Unadjusted cumulative incidence curves (CICs) of relapse according to the donor *IL6* SNP genotypes under the dominant model. Analyses included 766 malignant disease patients with no history of prior transplantation. Patients excluded for not achieving complete remission post-BMT totaled 56, and the primary competing events (relapse) numbered 152. The asterisk shows a significant difference (*p* < 0.0025 by Gray’s test).

In the univariable regression analysis, the *IL6* SNP, rs1800796, under the dominant model in donors was significantly associated with relapse, with CC being the risk genotype (SHR = 0.57; *p* = 0.0014 in Table 1). Consistent with this result, the CC genotype at this donor *IL6* SNP exhibited a significantly higher cumulative incidence of relapse (*p* = 0.0013; Figure 1). Donor rs1800796 under the additive model was also significantly associated with relapse (*p* = 0.0023 in Table 1). In the multivariable regression analysis, the donor *IL6* SNP under the dominant and additive models remained significant, with *p* = 0.0013 and 0.0023, respectively (Table 2).

### 3.5. Extensive Chronic GVHD (cGVHD)

In both univariable regression and multivariable regression analyses, no SNP was significantly associated with extensive cGVHD (Supplementary Tables S9 and S10). Among these SNPs, the donor *IL6* SNP under the dominant model in the univariable regression analysis exhibited the smallest *p*-value (*p* = 0.0038), in which CG + GG were the risk genotypes (SHR = 1.65) in contrast to relapse. This SNP was also not significant in the multivariable regression analysis (*p* = 0.014).

### 3.6. Other Outcomes

No correlations with these SNPs were identified for all (extensive + limited) cGVHD, NRM, or OS in the univariable or multivariable regression (Supplementary Tables S11–S16).

## 4. Discussion

In this study, we identified a significant association between the donor *IL6* gene SNP, rs1800796, (also referred to as -572C>G and -634C/G), and relapse of the primary disease (*p*~0.0013), with the CC genotype representing the risk variant (Figure 1 and Table 1). Although SNPs associated with relapse after allogeneic HSCT have been reported [39,40,41,42,43,44,45,46], this donor *IL6* SNP was also weakly associated with extensive cGVHD, with CC being the protective genotype (*p*~0.004). Since relapse and extensive cGVHD are competing events, donor *IL6* may play a direct role in relapse or extensive cGVHD, and, thus, may affect the rate of the other occurring. It is also possible that donor *IL6* affects both relapse and extensive cGVHD by changing the counteracting balance between the graft-versus-tumor and the graft-versus-host effects. In any case, consistent with the opposing relationships between the CC genotype and extensive cGVHD and relapse, this donor *IL6* SNP was not associated with OS. IL6 is a key proinflammatory cytokine released during the early phase of inflammation after conditioning [47] and has been implicated in both aGVHD and cGVHD [48]. Therefore, the donor *IL6* CC genotype, which was overrepresented in relapse and underrepresented in extensive cGVHD, may decrease the secretion of IL6 more than the CG and GG genotypes. However, since high levels of IL6 are associated with resistance to chemotherapy in AML and poor prognosis in pediatric AML [49,50], it is also possible that the donor *IL6* CC genotype exhibits an enhanced IL6 secretion. Additionally, previous studies show inconsistencies regarding whether this allele or genotype is associated with higher IL6 levels. Some studies reported that the C allele or CC genotype was associated with higher IL6 levels than the G allele or GG genotype in human serum, plasma, or cultured cells [51,52,53,54,55,56], while others, including one Japanese study, demonstrated the opposite [35,57,58,59,60,61,62,63], and some found no difference [64]. Therefore, it is unclear whether recipients with donor rs1800796 CC genotypes, which are associated with tumor relapse, secrete lower amounts of IL6. Rather than simply altering the levels of the encoded protein, an SNP may affect cancer susceptibility through other mechanisms, such as alterations of DNA modifications, histone modifications, and RNA splicing [65]. Although *IL6* SNPs have been associated with HSCT outcomes [9,12,18,24,27], to our knowledge, no HSCT studies have previously reported on this SNP, rs1800796.

The *IL2* -330 SNP (rs2069762) was not significantly associated with any outcome. However, the *p*-value for the relationship between this SNP in donors and grade 3–4 aGVHD was low (Supplementary Tables S7 and S8); therefore, further research involving larger sample sizes is needed. Findings of a recent research work indicated an association between recipient rs2069762 and NRM after single-unit cord blood transplant in 143 donor–recipient pairs [30]. Regarding the *IL2* -330 SNP (rs2069762), G allele carriers were found to produce more IL2 than T allele carriers [30,34,66], while another study reported opposite findings [67].

The *TGFB* SNP, rs1800469 (also referred to as -509C>T and -1347C>T), is located in a negative regulatory region of the promoter, and its A(T) allele has been shown to increase RNA transcription by preventing AP1 binding [37]. This *TGFB* SNP has been linked to coronary heart disease in a meta-analysis [68] and to heavy proteinuria and mesangial cell proliferation among Japanese patients with IgA nephropathy [69]. The *IFNG* SNP, rs2069705, also known as -1616T/C, is located in the 5′-UTR, and its A(T) allele is related to decreased levels of IFN-γ [36]. This *IFNG* SNP correlated (*p* = 0.0024) with systemic lupus erythematosus in a study involving 1759 Korean participants [70]. Therefore, these two *TGFB* and *IFNG* SNPs appear to be functional. The *TGFB* SNP has a high MAF, and, thus, the failure of this study to identify a correlation with BMT outcomes may be attributed to the genuine lack of a relationship.

The limitations of this study include its retrospective design. Clinical decisions need to be made based on prospective studies. The present results need to be validated in a large-scale study. Furthermore, the relationship between an SNP and disease is the potentially cumulative effect of many SNPs in linkage disequilibrium. Additionally, this study used the Seattle criteria [71], rather than the NIH criteria [72], for diagnosing cGVHD.

In conclusion, the donor *IL6* SNP, rs1800796 was associated with relapse of the primary disease but not with OS. If validated, this SNP may be useful for predicting relapse.

## Figures and Tables

**Table 1:** Univariable regressions of relapse on donor and recipient SNPs.

Gene	SNP (Donor/Recipient)	Additive Model		Dominant Model		Recessive Model	
		SHR (95% CI)	*p*	SHR (95% CI)	*p*	SHR (95% CI)	*p*
*IL2*	rs2069762 (d)	0.98 (0.77–1.24)	0.847	0.99 (0.72–1.36)	0.930	0.93 (0.54–1.59)	0.786
*IL6*	rs1800796 (d)	0.62 (0.45–0.84)	0.0023 *	0.57 (0.40–0.80)	0.0014 *	N.A. ^†^	N.A.
*IFNG*	rs2069765 (d)	0.96 (0.72–1.30)	0.803	0.99 (0.70–1.40)	0.965	N.A. ^†^	N.A.
*TGFB*	rs1800469 (d)	0.95 (0.77–1.17)	0.628	1.18 (0.81–1.73)	0.394	0.71 (0.46–1.08)	0.105
*IL2*	rs2069762 (r)	1.03 (0.80–1.31)	0.844	0.98 (0.71–1.34)	0.895	1.18 (0.71–1.96)	0.521
*IL6*	rs1800796 (r)	1.12 (0.86–1.45)	0.403	1.08 (0.78–1.48)	0.651	N.A. ^†^	N.A.
*IFNG*	rs2069765 (r)	1.18 (0.87–1.59)	0.290	1.23 (0.87–1.72)	0.243	N.A. ^†^	N.A.
*TGFB*	rs1800469 (r)	0.88 (0.68–1.13)	0.308	0.68 (0.49–0.96)	0.029	1.09 (0.75–1.57)	0.666

The outcomes for each SNP were determined through separate regressions analyses conducted under the three specified genetic models. The analysis included 766 malignant disease patients without prior transplantation history, excluding 56 cases lacking complete remission post-BMT. Primary competing events (relapse) totaled 152. Refer to Table 1 legend for additional details. ^†^ Not applicable (N.A.) for low minor allele frequency. * *p* < 0.0025 by the Wald test.

**Table 2:** Multivariable regressions of relapse on donor *IL6* rs1800796 under the additive and dominant models, adjusted by reported risk factors.

	Additive Model		Dominant Model	
	SHR (95% CI)	*p*	SHR (95% CI)	*p*
Donor *IL6* rs1800796	0.61 (0.44–0.84)	0.0023 *	0.56 (0.39–0.79)	0.0013 *
HLA-C mismatch	0.78 (0.53–1.16)	0.227	0.78 (0.53–1.16)	0.220
ABO mismatch	1.20 (0.87–1.66)	0.270	1.20 (0.86–1.66)	0.277
Donor CMV status ^§^	1.86 (1.23–2.82)	0.0035	1.87 (1.23–2.83)	0.0033
Performance status, high vs. low	1.22 (0.88–1.69)	0.229	1.22 (0.88–1.69)	0.237
Disease stage ^†^	1.66 (1.19–2.32)	0.0031	1.66 (1.19–2.32)	0.0031
Recipient age, high vs. low	0.84 (0.60–1.17)	0.299	0.84 (0.60–1.17)	0.295
Donor age, high vs. low	0.99 (0.72–1.38)	0.963	0.99 (0.71–1.37)	0.952
Female donor–male recipient ^‡^	1.00 (0.66–1.52)	0.996	1.00 (0.66–1.52)	0.997

“Additive model” in the top row of this Table denotes that donor *IL6* rs1800796 SNP in the first row is under the additive model (GG vs. CG vs. CC), whereas “Dominant model” denotes that the donor *IL6* rs1800796 SNP is under the dominant model (CG + GG vs. CC). The analysis included 766 malignant disease patients without prior transplantation history, excluding 56 cases lacking complete remission post-BMT. Primary competing events (relapse) totaled 152. Refer to Table 1’s legend for additional details. While HLA-C mismatches are used in these regressions, the results obtained did not qualitatively change when total HLA mismatches were used. ^§^ Yes + unknown vs. no. ^†^ Advanced + unknown vs. standard. ^‡^ vs. female donor–female recipient, male donor–male recipient, and male donor–female recipient, combined. * *p* < 0.0025 by the Wald test.

## Data Availability

Data are available from the corresponding author upon reasonable request with the permission of the JMDP/JDCHCT.

## Supplementary Materials

The following supporting information can be downloaded at https://www.mdpi.com/article/10.3390/jcm14020476/s1: Table S1. Characteristics of 822 malignant disease patients without a transplantation history and their unrelated BMT donor; Table S2. SNP information; Table S3. Summary of genotyping of 999 donors and 999 recipients; Table S4. SNP frequency; Table S5. Univariable regression of grade 2–4 aGVHD on donor and recipient SNPs; Table S6. Multivariable regression of grade 2–4 aGVHD on donor and recipient SNPs; Table S7. Univariable regression of grade 3–4 aGVHD on donor and recipient SNPs; Table S8. Multivariable regression of grade 3–4 aGVHD on donor and recipient SNPs; Table S9. Univariable regression of extensive cGVHD on donor and recipient SNPs; Table S10. Multivariable regression of extensive cGVHD on donor and recipient SNPs; Table S11. Univariable regression of all cGVHD on donor and recipient SNPs; Table S12. Multivariable regression of all cGVHD on donor and recipient SNPs; Table S13. Univariable regression of non-relapse mortality on donor and recipient SNPs; Table S14. Multivariable regression of non-relapse mortality on donor and recipient SNPs; Table S15. Univariable regression of overall survival on donor and recipient SNPs; Table S16. Multivariable regression of overall survival on donor and recipient SNPs.
