# Supplementary material for: Relationship Between an Interleukin 6 SNP and Relapse After Allogeneic Bone Marrow Transplantation
Source: J Clin Med. 2025 Jan 13;14(2):476. doi: 10.3390/jcm14020476 (PMC11765773; doi:10.3390/jcm14020476)
Supplement: Supplementary file 1 [file jcm-14-00476-s001.zip › jcm-3342547-supplementary.pdf]

Supplementary Information for

Relationship between an *interleukin 6* SNP and relapse after allogeneic bone marrow transplantation

Hidekazu Takahashi[1], Natsu Yamaguchi[1], Naoko Okayama[2,3], Mitsuaki Nishioka[2], MH Mahbub[1], Ryosuke Hase[1], Yutaka Suehiro[2,3,4], Takahiro Yamasaki[2,4], Satoshi Takahashi[5], Arinobu Tojo[6] and Tsuyoshi Tanabe[1,*]

[1]Department of Public Health and Preventive Medicine, Yamaguchi University Graduate School of Medicine, Ube, Japan.

[2]Division of Laboratory, Yamaguchi University Hospital, Ube, Japan.

[3]Division of Medical Genetics, Yamaguchi University Hospital, Ube, Japan.

[4]Department of Oncology and Laboratory Medicine, Yamaguchi University Graduate School of Medicine, Ube, Japan.

[5]Division of Clinical Precision Research Platform, the Institute of Medical Science, The University of Tokyo, Tokyo, Japan.

[6]Project Professor, Tokyo Medical and Dental University, Tokyo, Japan.

*Correspondence: tanabe@yamaguchi-u.ac.jp.

Table of Contents

Content **Pages**

Table S1. Characteristics of 822 malignant disease patients without a transplantation history and their unrelated BMT donor 3

Table S2. SNP information 4

Table S3. Summary of genotyping of 999 donors and 999 recipients 5

Table S4. SNP frequency 6

Table S5. Univariable regression of grade 2–4 aGVHD on donor and recipient SNPs 7

Table S6. Multivariable regression of grade 2–4 aGVHD on donor and recipient SNPs 8

Table S7. Univariable regression of grade 3–4 aGVHD on donor and recipient SNPs 9

Table S8. Multivariable regression of grade 3–4 aGVHD on donor and recipient SNPs 10

Table S9. Univariable regression of extensive cGVHD on donor and recipient SNPs 11

Table S10. Multivariable regression of extensive cGVHD on donor and recipient SNPs 12

Table S11. Univariable regression of all cGVHD on donor and recipient SNPs 13

Table S12. Multivariable regression of all cGVHD on donor and recipient SNPs 14

Table S13. Univariable regression of non-relapse mortality on donor and recipient SNPs 15

Table S14. Multivariable regression of non-relapse mortality on donor and recipient SNPs 16

Table S15. Univariable regression of overall survival on donor and recipient SNPs 17

Table S16. Multivariable regression of overall survival on donor and recipient SNPs 18

Table S1. Characteristics of 822 malignant disease patients without a transplantation history and their unrelated BMT donor

**Recipient characteristics**	***N* = 822**
Sex	
Female	336
Male	486
Age (year)	
Low (1–44)	379
High (45–71)	443
Underlying disease	
Acute myeloid leukemia (AML)	342
Acute lymphoblastic leukemia (ALL)	178
Myelodysplastic syndromes (MDS)	120
Chronic myeloid leukemia (CML)	27
Other leukemia	66
Lymphoid malignancy (LM)	76
Myeloproliferative disorders (MPD)	8
Plasma cell dyscrasias (PCD)	3
Solid tumors (ST)	2
Disease stage	
Standard	450
Advanced or unknown	372
Body mass index (BMI) (kg/m^2)	
Low (12.0–22.0) or unknown	469
High (22.0–38.6)	353
CMV serostatus before BMT	
Negative	141
Positive or unknown	681
Performance status (PS) before BMT	
Low (0)	478
High (1–4)	344

**Donor characteristics**	***N* = 822**
Sex	
Female	260
Male	562
Age	
Low (20–33 years)	396
High (34–68 years)	426
CMV serostatus before BMT	
Negative	253
Positive or unknown	569

**Unrelated BMT characteristics**	***N* = 822**
Myeloablative conditioning	
No	177
Yes	645
Cyclosporine A (CyA)‖	
No or unknown	597
Yes	225
Ara-C	
No	731
Yes	91
Cyclophosphamide	
No	354
Yes	468
N of nucleated cells infused¶	
Low (0.01-2.50) or unknown	430
High (≥2.50)	392
Days from diagnosis to BMT	
Low (9-269)	415
High (270-10897) or unknown	407
ABO blood type	
Match	464
Mismatch	358
HLA-C	
0 mismatches	636
1 mismatch (both directions)	147
1 mismatch (GvH direction)	9
1 mismatch (rejection direction)	16
2 mismatches (both directions)	14
HLA-DQB1	
0 mismatches	758
1 mismatch (both directions)	52
1 mismatch (GvH direction)	5
1 mismatch (rejection direction)	7
2 mismatches (both directions)	0
HLA-DPB1	
0 mismatches	195
1 mismatch (both directions)	331
1 mismatch (GvH direction)	68
1 mismatch (rejection direction)	68
2 mismatches (both directions)	160
HLA-C, -DQB1 or -DPB1 (GvH)	
0 mismatches	214
1 mismatch (GvH direction)	330
2 mismatches (GvH direction)	211
3 mismatches (GvH direction)	60
4 mismatches (GvH direction)	7
HLA-C, -DQB1 or -DPB1 (bi-directional)	
0 mismatches	155
1 mismatch (either direction)	369
2 mismatches (either direction)	223
3 mismatches (either direction)	64
4 mismatches (either direction)	11

These 822 pairs are identical to Group 1 described in Supplementary Table S1 of Takahashi et al. [15].

See [15] for details of these characteristics. The HSC source was exclusively from bone marrow.

‖ Tacrolimus was administered to 594 of the 596 CyA non-users and to 6 of the 225 CyA users. ¶10^8 per kg body weight.

Table S2. SNP information

SNP	Alternative name	Gene	SNP type	Chromosome	Location in GRCh37*	Location in GRCh38*	Allele 1†	Allele 2†
rs2069762	-330	*IL2*	Upstream	4	123377980	122456825	T	G
rs1800796	-572(-634)	*IL6*	Upstream	7	22766246	22726627	C	G
rs2069705	-1616T/C	*IFNG*	Upstream	12	68555011	68161231	G	A
rs1800469	-509C>T or -1347C>T	*TGFB*	Upstream	19	41860296	41354391	G	A

SNP	Reported molecular function of allele 2 relative to allele 1	DNA sequence around the SNP
rs2069762	PBL from healthy volunteers with the GG genotype exhibited increased IL2 production due to an early and sustained enhancement [34]	AAAATTTTCTTTGTC[**C/A**]TAAAACTACACTGAA
rs1800796	Individuals carrying the -634 G allele had a higher IL-6 secretion capacity by PMBC, when stimulated by LPS and by AGE-BSA, than those without the -634 G allele [35]	AGTTCTACAACAGCC[**C/G**]CTCACAGGGAGAGCC
rs2069705	The A allele has been associated with a lower level of IFN-γ [36]	TATGATTGTGAGTTA[**A/G**]CTTCTTAAATCTTCT
rs1800469	The A allele increased transcription by preventing AP1 binding [37]	CAACAGGACACCTGA[**A/G**]GGATGGAAGGGTCAG

SNP	TaqMan Assay ID	PCR primer A (5' -> 3')	PCR primer B (5' -> 3')
rs2069762	C__15859930_10	CCACACTTAGGTGATAGCTCTAATTCATG	CTTTTCATCTGTTTACTCTTGCTCTTGTC
rs1800796	C__11326893_10	CCAAAGATGTTCTGAACTGAGTTTCC	CAAAAAGGAGTCACACACTCCACC
rs2069705	C__15944115_20	CCCTCCACTCTTTGGTTCAAACC	CCTAGCACTTTATGAGGATTACCTG
rs1800469	C___8708473_10	GTCGCAGGGTGTTGAGTGACAG	GAGTCAGGCTGGGAAACAAGGTAG

TaqMan assays and the oligo DNA sequences used for direct DNA sequencing are shown.
*GRCh37 and 38 stand for Genome Reference Consortium human genome build 37 and build 38, respectively.
†Alleles 1 and 2 exhibit variant nucleotides

Table S3. Summary of genotyping of 999 donors and 999 recipients

Gene	SNP	N of subjects successfully genotyped in one round of the TaqMan assay	N of subjects successfully genotyped by PCR direct sequencing after a failure in the TaqMan assay	N of subjects genotyped only by PCR direct sequencing	N of subjects with the final genotype undetermined
IL2	rs2069762	1904	68	26	0
IL6	rs1800796	1925	47	26	0
IFNG	rs2069765	1958	14	26	0
TGFB	rs1800469	1924	48	26	0

Table S4. SNP frequency

	All first-time recipients (*n* = 887)	All first-time donors (*n* = 887)	JPT104 (*n* = 104)
rs2069762 (*IL2*)			
AA	419	394	54
AC	375	392	44
CC	93	101	6
C allele frequency	0.316	0.335	0.269
p for HWE	0.534	0.821	0.618
rs1800796 (*IL6*)			
CC	546	533	70
CG	290	305	31
GG	51	49	3
G allele frequency	0.221	0.227	0.178
p for HWE	0.143	0.566	1.000
rs2069765 (*IFNG*)			
GG	644	620	82
AG	225	245	21
AA	18	22	1
A allele frequency	0.147	0.163	0.111
p for HWE	0.893	0.806	1.000
rs1800469 (*TGFB*)			
AA	234	228	29
AG	451	467	52
GG	202	192	23
G allele frequency	0.482	0.480	0.471
p for HWE	0.638	0.122	1.000

Genotype frequency, minor allele frequency, and the Hardy–Weinberg equilibrium (HWE) of SNPs in all (including non-malignant disease) first-time transplantation recipients and their donors (Groups 1+2 in Supplementary Table S1 of Takahashi et al. [15]), and in Japanese residents of Tokyo (JPT104) in the 1000 Genomes Project [33] are shown. No significant violation of HWE was observed (statistical significance was determined by the exact test).

Table S5. Univariable regression of grade 2–4 aGVHD on donor and recipient SNPs

Gene	SNP (donor /recipient)	Additive model		Dominant model		Recessive model	
		SHR (95% CI)	*p*	SHR (95% CI)	*p*	SHR (95% CI)	*p*
IL2	rs2069762 (d)	0.80 (0.67–0.96)	.018	0.77 (0.61–0.97)	.026	0.73 (0.49–1.10)	.129
IL6	rs1800796 (d)	1.14 (0.95–1.38)	.160	1.12 (0.88–1.41)	.354	N.A.†	N.A.
IFNG	rs2069765 (d)	1.04 (0.84–1.28)	.743	1.06 (0.83–1.36)	.639	N.A.†	N.A.
TGFB	rs1800469 (d)	0.94 (0.79–1.10)	.433	0.97 (0.75–1.26)	.823	0.86 (0.64–1.15)	.297
IL2	rs2069762 (r)	0.95 (0.80–1.14)	.584	0.88 (0.70–1.12)	.297	1.09 (0.77–1.56)	.627
IL6	rs1800796 (r)	1.01 (0.83–1.23)	.931	1.00 (0.79–1.27)	.995	N.A.†	N.A.
IFNG	rs2069765 (r)	1.06 (0.84–1.33)	.613	1.09 (0.84–1.41)	.536	N.A.†	N.A.
TGFB	rs1800469 (r)	1.08 (0.91–1.26)	.382	1.23 (0.93–1.63)	.156	0.99 (0.75–1.30)	.946

The results for each SNP were obtained by running separate subdistribution hazard regressions under the three genetic models indicated in the top row of the table. Malignant disease patients without a previous transplantation history were analyzed (n = 787). Excluded: aGVHD unevaluable (n = 34) and the day of grade 2/3/4 aGVHD unknown (n = 1). The number of primary competing events (grade 2–4 aGVHD) = 280. In the second column of the table, (d) and (r) stand for donor and recipient, respectively. SHR, subdistribution hazard ratio; CI, confidence interval. †Not applicable (N.A.) due to a low minor allele frequency. No SNP correlated with grade 2–4 aGVHD (statistical significance was determined using the Wald test).

Table S6. Multivariable regression of grade 2–4 aGVHD on donor and recipient SNPs

Gene	SNP (donor /recipient)	Additive model		Dominant model		Recessive model	
		SHR (95% CI)	*p*	SHR (95% CI)	*p*	SHR (95% CI)	*p*
IL2	rs2069762 (d)	0.80 (0.66–0.96)	.015	0.76 (0.60–0.96)	.020	0.72 (0.48–1.10)	.134
IL6	rs1800796 (d)	1.18 (0.98–1.42)	.087	1.17 (0.92–1.49)	.201	N.A.†	N.A.
IFNG	rs2069765 (d)	1.03 (0.83–1.28)	.814	1.05 (0.82–1.35)	.706	N.A.†	N.A.
TGFB	rs1800469 (d)	0.93 (0.78–1.10)	.386	0.93 (0.71–1.21)	.571	0.88 (0.66–1.19)	.412
IL2	rs2069762 (r)	0.94 (0.78–1.13)	.507	0.88 (0.69–1.11)	.274	1.06 (0.74–1.52)	.759
IL6	rs1800796 (r)	1.01 (0.83–1.23)	.915	1.01 (0.79–1.28)	.959	N.A.†	N.A.
IFNG	rs2069765 (r)	1.07 (0.85–1.34)	.561	1.09 (0.84–1.42)	.503	N.A.†	N.A.
TGFB	rs1800469 (r)	1.05 (0.89–1.24)	.586	1.15 (0.87–1.53)	.327	0.98 (0.74–1.30)	.909

The results for each SNP were obtained by running separate subdistribution hazard regressions under the three genetic models indicated in the top row of the table, adjusted with total HLA mismatches, CyA, BMI, myeloablative, disease stage, recipient age, donor age, and female donor–male recipient. Malignant disease patients without a previous transplantation history were analyzed (n = 787). Excluded: aGVHD unevaluable (n = 34) and the day of grade 2/3/4 aGVHD unknown (n = 1). The number of primary competing events (grade 2–4 aGVHD) = 280. In the second column of the table, (d) and (r) stand for donor and recipient, respectively. SHR, subdistribution hazard ratio; CI, confidence interval. †Not applicable (N.A.) due to a low minor allele frequency. No SNP correlated with grade 2–4 aGVHD (statistical significance was determined using the Wald test).

Table S7. Univariable regression of grade 3–4 aGVHD on donor and recipient SNPs

Gene	SNP (donor /recipient)	Additive model		Dominant model		Recessive model	
		SHR (95% CI)	*p*	SHR (95% CI)	*p*	SHR (95% CI)	*p*
IL2	rs2069762 (d)	0.59 (0.40–0.85)	.0052	0.52 (0.33–0.80)	**.0035**	0.52 (0.21–1.27)	.151
IL6	rs1800796 (d)	0.98 (0.68–1.42)	.917	0.94 (0.60–1.47)	.794	N.A.†	N.A.
IFNG	rs2069765 (d)	0.78 (0.49–1.25)	.304	0.74 (0.44–1.22)	.239	N.A.†	N.A.
TGFB	rs1800469 (d)	0.99 (0.73–1.33)	.931	1.18 (0.70–1.99)	.543	0.80 (0.46–1.40)	.440
IL2	rs2069762 (r)	0.86 (0.62–1.18)	.344	0.88 (0.57–1.37)	.578	0.64 (0.28–1.46)	.288
IL6	rs1800796 (r)	0.99 (0.69–1.42)	.950	1.01 (0.64–1.58)	.967	N.A.†	N.A.
IFNG	rs2069765 (r)	0.82 (0.53–1.27)	.366	0.87 (0.53–1.44)	.588	N.A.†	N.A.
TGFB	rs1800469 (r)	1.02 (0.78–1.34)	.864	1.52 (0.87–2.68)	.142	0.69 (0.39–1.22)	.200

The results for each SNP were obtained by running separate regressions under the three genetic models, indicated in the top row of the table. Malignant disease patients without a previous transplantation history were analyzed (n = 787). Excluded: aGVHD unevaluable (n = 34) and the day of grade 2/3/4 aGVHD unknown (n = 1). The number of primary competing events (grade 3–4 aGVHD) = 80. In the second column of the table, (d) and (r) stand for donor and recipient, respectively. SHR, subdistribution hazard ratio; CI, confidence interval.

†Not applicable (N.A.) due to a low minor allele frequency.

No SNP correlated with grade 2–4 aGVHD (statistical significance was determined using the Wald test). The donor *IL2* SNP in the dominant model exhibited the lowest *p*-value (p = 0.0035; shown in bold letters).

Table S8. Multivariable regression of grade 3–4 aGVHD on donor and recipient SNPs

Gene	SNP (donor /recipient)	Additive model		Dominant model		Recessive model	
		SHR (95% CI)	*p*	SHR (95% CI)	*p*	SHR (95% CI)	*p*
IL2	rs2069762 (d)	0.59 (0.41–0.87)	.0071	0.52 (0.33–0.81)	**.0042**	0.55 (0.23–1.35)	.193
IL6	rs1800796 (d)	1.01 (0.70–1.46)	.956	0.99 (0.63–1.57)	.967	N.A.†	N.A.
IFNG	rs2069765 (d)	0.78 (0.49–1.25)	.307	0.74 (0.45–1.23)	.244	N.A.†	N.A.
TGFB	rs1800469 (d)	0.97 (0.72–1.32)	.852	1.10 (0.65–1.84)	.724	0.83 (0.48–1.45)	.519
IL2	rs2069762 (r)	0.82 (0.59–1.15)	.248	0.85 (0.54–1.34)	.484	0.59 (0.26–1.35)	.210
IL6	rs1800796 (r)	0.99 (0.70–1.42)	.970	1.02 (0.65–1.59)	.931	N.A.†	N.A.
IFNG	rs2069765 (r)	0.79 (0.51–1.24)	.312	0.84 (0.50–1.40)	.504	N.A.†	N.A.
TGFB	rs1800469 (r)	1.00 (0.75–1.33)	.995	1.44 (0.81–2.56)	.215	0.68 (0.38–1.22)	.201

The results for each SNP were obtained by running separate subdistribution hazard regressions under the three genetic models indicated in the top row of the table, adjusted with total HLA mismatches, CyA, BMI, myeloablative, disease stage, recipient age, donor age, and female donor–male recipient. Malignant disease patients without a previous transplantation history were analyzed (n = 787). Excluded: aGVHD unevaluable (n = 34) and the day of grade 2/3/4 aGVHD unknown (n = 1). The number of primary competing events (grade 2–4 aGVHD) = 80. In the second column of the table, (d) and (r) stand for donor and recipient, respectively. SHR, subdistribution hazard ratio; CI, confidence interval. †Not applicable (N.A.) due to a low minor allele frequency. No SNP correlated with grade 2–4 aGVHD (statistical significance was determined using the Wald test). The donor *IL2* SNP in the dominant model exhibited the lowest p-value (p = 0.0042; shown in bold letters).

Table S9. Univariable regression of extensive cGVHD on donor and recipient SNPs

Gene	SNP (donor /recipient)	Additive model		Dominant model		Recessive model	
		**SHR (95% CI)**	***p***	**SHR (95% CI)**	***p***	**SHR (95% CI)**	***p***
IL2	rs2069762 (d)	1.06 (0.82–1.37)	.660	1.05 (0.75–1.49)	.772	1.14 (0.68–1.90)	.630
IL6	rs1800796 (d)	1.36 (1.06–1.74)	.016	1.65 (1.18–2.33)	**.0038**	N.A.†	N.A.
IFNG	rs2069765 (d)	1.20 (0.87–1.65)	.277	1.16 (0.81–1.67)	.420	N.A.†	N.A.
TGFB	rs1800469 (d)	0.98 (0.76–1.26)	.859	0.91 (0.62–1.34)	.629	1.04 (0.70–1.55)	.834
IL2	rs2069762 (r)	1.09 (0.85–1.40)	.507	1.03 (0.73–1.45)	.873	1.34 (0.84–2.15)	.225
IL6	rs1800796 (r)	0.80 (0.59–1.08)	.142	0.79 (0.55–1.13)	.200	N.A.†	N.A.
IFNG	rs2069765 (r)	0.99 (0.71–1.38)	.955	1.02 (0.70–1.48)	.918	N.A.†	N.A.
TGFB	rs1800469 (r)	1.20 (0.95–1.53)	.128	1.45 (0.95–2.23)	.087	1.14 (0.76–1.71)	.521

The results for each SNP were obtained by running separate regressions under the three genetic models, indicated in the top row of the table. Malignant disease patients without a previous transplantation history were analyzed (n = 677). Excluded: cGVHD unevaluable (n = 142) and the day of cGVHD unknown (n = 3). The number of primary competing events (extensive cGVHD) = 132. In the second column of the table, (d) and (r) stand for donor and recipient, respectively. SHR, subdistribution hazard ratio; CI, confidence interval. †Not applicable (N.A.) due to a low minor allele frequency. No SNP correlated with extensive cGVHD (statistical significance was determined using the Wald test). The donor *IL6* SNP in the dominant model exhibited the lowest p-value (p = 0.0038; shown in bold letters).

Table S10. Multivariable regression of extensive cGVHD on donor and recipient SNPs

Gene	SNP (donor /recipient)	Additive model		Dominant model		Recessive model	
		SHR (95% CI)	*p*	SHR (95% CI)	*p*	SHR (95% CI)	*p*
IL2	rs2069762 (d)	1.11 (0.86–1.43)	.416	1.13 (0.80–1.60)	.475	1.17 (0.69–1.99)	.552
IL6	rs1800796 (d)	1.29 (1.00–1.68)	.053	1.54 (1.09–2.19)	.014	N.A.†	N.A.
IFNG	rs2069765 (d)	1.24 (0.90–1.71)	.185	1.24 (0.86–1.80)	.245	N.A.†	N.A.
TGFB	rs1800469 (d)	1.03 (0.80–1.31)	.833	1.03 (0.69–1.54)	.873	1.04 (0.69–1.56)	.852
IL2	rs2069762 (r)	1.05 (0.81–1.36)	.726	0.95 (0.67–1.34)	.758	1.36 (0.86–2.17)	.191
IL6	rs1800796 (r)	0.88 (0.65–1.21)	.437	0.88 (0.60–1.27)	.486	N.A.†	N.A.
IFNG	rs2069765 (r)	1.01 (0.73–1.40)	.961	1.07 (0.73–1.56)	.733	N.A.†	N.A.
TGFB	rs1800469 (r)	1.20 (0.94–1.54)	.135	1.46 (0.95–2.26)	.086	1.14 (0.76–1.71)	.540

The results for each SNP were obtained by running separate subdistribution hazard regressions under the three genetic models indicated in the top row of the table, adjusted with HLA-C mismatch, BMI, myeloablative, disease stage, recipient age, donor age, and female donor–male recipient. Malignant disease patients without a previous transplantation history were analyzed (n = 677). Excluded: cGVHD unevaluable (n = 142) and the day of cGVHD unknown (n = 3). The number of primary competing events (extensive cGVHD) = 132. In the second column of the table, (d) and (r) stand for donor and recipient, respectively. SHR, subdistribution hazard ratio; CI, confidence interval. †Not applicable (N.A.) due to a low minor allele frequency. No SNP correlated with extensive cGVHD (statistical significance was determined using the Wald test).

Table S11. Univariable regression of all cGVHD on donor and recipient SNPs

Gene	SNP (donor /recipient)	Additive model		Dominant model		Recessive model	
		SHR (95% CI)	*p*	SHR (95% CI)	*p*	SHR (95% CI)	*p*
IL2	rs2069762 (d)	1.04 (0.86–1.25)	.680	1.08 (0.83–1.39)	.582	1.00 (0.67–1.48)	.987
IL6	rs1800796 (d)	1.17 (0.96–1.42)	.115	1.37 (1.06–1.77)	.016	N.A.†	N.A.
IFNG	rs2069765 (d)	1.05 (0.82–1.34)	.705	1.05 (0.79–1.38)	.751	N.A.†	N.A.
TGFB	rs1800469 (d)	0.99 (0.82–1.20)	.894	0.90 (0.67–1.20)	.468	1.09 (0.81–1.46)	.588
IL2	rs2069762 (r)	1.01 (0.84–1.22)	.933	0.99 (0.77–1.27)	.927	1.07 (0.72–1.57)	.744
IL6	rs1800796 (r)	0.93 (0.76–1.15)	.515	0.97 (0.75–1.26)	.820	N.A.†	N.A.
IFNG	rs2069765 (r)	1.07 (0.83–1.38)	.585	1.04 (0.78–1.38)	.786	N.A.†	N.A.
TGFB	rs1800469 (r)	1.17 (0.98–1.40)	.089	1.37 (1.00–1.88)	.051	1.12 (0.83–1.51)	.473

The results for each SNP were obtained by running separate regressions under the three genetic models, indicated in the top row of the table. Malignant disease patients without a previous transplantation history were analyzed (n = 677). Excluded: cGVHD unevaluable (n = 142) and the day of cGVHD unknown (n = 3). The number of primary competing events (limited + extensive cGVHD) = 235. In the second column of the table, (d) and (r) stand for donor and recipient, respectively. SHR, subdistribution hazard ratio; CI, confidence interval. †Not applicable (N.A.) due to a low minor allele frequency. No SNP correlated with all cGVHD (statistical significance was determined using the Wald test).

Table S12. Multivariable regression of all cGVHD on donor and recipient SNPs

Gene	SNP (donor /recipient)	Additive model		Dominant model		Recessive model	
		SHR (95% CI)	*p*	SHR (95% CI)	*p*	SHR (95% CI)	*p*
IL2	rs2069762 (d)	1.04 (0.86–1.26)	.693	1.07 (0.83–1.39)	.592	1.00 (0.66–1.50)	.990
IL6	rs1800796 (d)	1.14 (0.94–1.39)	.186	1.31 (1.02–1.70)	.036	N.A.†	N.A.
IFNG	rs2069765 (d)	1.09 (0.86–1.39)	.482	1.10 (0.84–1.46)	.484	N.A.†	N.A.
TGFB	rs1800469 (d)	1.00 (0.83–1.21)	.965	0.96 (0.72–1.29)	.799	1.05 (0.77–1.44)	.736
IL2	rs2069762 (r)	0.98 (0.81–1.19)	.866	0.94 (0.72–1.22)	.641	1.00 (0.66–1.50)	.990
IL6	rs1800796 (r)	0.99 (0.80–1.23)	.941	1.03 (0.79–1.34)	.847	N.A.†	N.A.
IFNG	rs2069765 (r)	1.08 (0.85–1.37)	.545	1.07 (0.81–1.42)	.640	N.A.†	N.A.
TGFB	rs1800469 (r)	1.16 (0.97–1.39)	.099	1.39 (1.02–1.91)	.040	1.09 (0.80–1.47)	.590

The results for each SNP were obtained by running separate subdistribution hazard regressions under the three genetic models indicated in the top row of the table, adjusted with HLA-C mismatch, BMI, myeloablative, disease stage, recipient age, donor age, and female donor–male recipient. Malignant disease patients without a previous transplantation history were analyzed (n = 677). Excluded: cGVHD unevaluable (n = 142) and the day of cGVHD unknown (n = 3). The number of primary competing events (limited + extensive cGVHD) = 235. In the second column of the table, (d) and (r) stand for donor and recipient, respectively. SHR, subdistribution hazard ratio; CI, confidence interval. †Not applicable (N.A.) due to a low minor allele frequency. No SNP correlated with all cGVHD (statistical significance was determined using the Wald test).

Table S13. Univariable regression of non-relapse mortality on donor and recipient SNPs

Gene	SNP (donor /recipient)	Additive model		Dominant model		Recessive model	
		SHR (95% CI)	*p*	SHR (95% CI)	*p*	SHR (95% CI)	*p*
IL2	rs2069762 (d)	0.96 (0.77–1.21)	.750	0.94 (0.70–1.25)	.656	1.01 (0.62–1.63)	.980
IL6	rs1800796 (d)	1.09 (0.85–1.39)	.512	1.02 (0.76–1.37)	.871	N.A.†	N.A.
IFNG	rs2069765 (d)	0.96 (0.71–1.30)	.794	0.89 (0.64–1.23)	.465	N.A.†	N.A.
TGFB	rs1800469 (d)	1.30 (1.05–1.61)	.016	1.34 (0.95–1.91)	.099	1.47 (1.06–2.03)	.021
IL2	rs2069762 (r)	0.88 (0.71–1.10)	.255	0.84 (0.63–1.13)	.245	0.87 (0.54–1.39)	.554
IL6	rs1800796 (r)	0.93 (0.73–1.18)	.549	0.98 (0.73–1.32)	.896	N.A.†	N.A.
IFNG	rs2069765 (r)	0.80 (0.59–1.08)	.143	0.80 (0.57–1.12)	.200	N.A.†	N.A.
TGFB	rs1800469 (r)	1.09 (0.89–1.34)	.411	1.11 (0.79–1.55)	.561	1.14 (0.82–1.59)	.442

The results for each SNP were obtained by running separate subdistribution hazard regressions under the three genetic models indicated. Malignant disease patients without a previous transplantation history were analyzed (*n* = 766). Excluded: no complete remission achieved after BMT (*n* = 56). The number of primary competing events (non-relapse mortality) = 182. In the second column of the table, (d) and (r) stand for donor and recipient, respectively. SHR, subdistribution hazard ratio; CI, confidence interval. †Not applicable (N.A.) due to a low minor allele frequency. No SNP correlated with non-relapse mortality (statistical significance was determined using the Wald test).

Table S14. Multivariable regression of non-relapse mortality on donor and recipient SNPs

Gene	SNP (donor /recipient)	Additive model		Dominant model		Recessive model	
		SHR (95% CI)	*p*	SHR (95% CI)	*p*	SHR (95% CI)	*p*
IL2	rs2069762 (d)	0.98 (0.78–1.23)	.885	0.98 (0.73–1.31)	.887	0.98 (0.59–1.62)	.933
IL6	rs1800796 (d)	1.09 (0.85–1.40)	.494	1.04 (0.78–1.40)	.783	N.A.†	N.A.
IFNG	rs2069765 (d)	0.97 (0.72–1.31)	.827	0.90 (0.65–1.24)	.512	N.A.†	N.A.
TGFB	rs1800469 (d)	1.29 (1.04–1.60)	.022	1.31 (0.92–1.88)	.133	1.46 (1.05–2.02)	.024
IL2	rs2069762 (r)	0.85 (0.68–1.06)	.148	0.80 (0.60–1.07)	.135	0.85 (0.53–1.34)	.477
IL6	rs1800796 (r)	0.99 (0.78–1.25)	.908	1.05 (0.78–1.42)	.734	N.A.†	N.A.
IFNG	rs2069765 (r)	0.84 (0.62–1.14)	.255	0.86 (0.61–1.21)	.380	N.A.†	N.A.
TGFB	rs1800469 (r)	1.09 (0.88–1.35)	.444	1.11 (0.78–1.58)	.552	1.12 (0.80–1.59)	.507

The results for each SNP were obtained by running separate subdistribution hazard regressions under the three genetic models indicated in the top row of the table, adjusted with HLA-C mismatch, ABO mismatch, donor CMV status, recipient performance status, disease stage, recipient age, donor age, and female donor–male recipient. Malignant disease patients without a previous transplantation history were analyzed (n = 766). Excluded: no complete remission achieved after BMT (n = 56). The number of primary competing events (non-relapse mortality) = 182. In the second column of the table, (d) and (r) stand for donor and recipient, respectively. SHR, subdistribution hazard ratio; CI, confidence interval. †Not applicable (N.A.) due to a low minor allele frequency. No SNP correlated with non-relapse mortality (statistical significance was determined using the Wald test).

Table S15. Univariable regression of overall survival on donor and recipient SNPs

Gene	SNP (donor /recipient)	Additive model		Dominant model		Recessive model	
		HR (95% CI)	*p*	HR (95% CI)	*p*	HR (95% CI)	*p*
IL2	rs2069762 (d)	0.96 (0.82–1.12)	.574	0.90 (0.73–1.11)	.343	1.06 (0.76–1.47)	.749
IL6	rs1800796 (d)	0.90 (0.76–1.08)	.276	0.84 (0.68–1.04)	.117	N.A.†	N.A.
IFNG	rs2069765 (d)	1.08 (0.88–1.31)	.469	1.06 (0.84–1.32)	.629	N.A.†	N.A.
TGFB	rs1800469 (d)	1.11 (0.96–1.29)	.159	1.23 (0.96–1.58)	.100	1.08 (0.84–1.38)	.546
IL2	rs2069762 (r)	0.95 (0.81–1.11)	.526	0.92 (0.75–1.13)	.422	0.99 (0.71–1.38)	.935
IL6	rs1800796 (r)	1.02 (0.86–1.21)	.848	1.01 (0.82–1.25)	.900	N.A.†	N.A.
IFNG	rs2069765 (r)	0.82 (0.65–1.02)	.071	0.81 (0.63–1.03)	.084	N.A.†	N.A.
TGFB	rs1800469 (r)	0.98 (0.84–1.14)	.810	0.83 (0.66–1.05)	.122	1.16 (0.91–1.48)	.224

The results for each SNP were obtained by running separate Cox regressions under the three genetic models, indicated in the top row of the table. Malignant disease patients without a previous transplantation history were analyzed (n = 822). The number of primary events (death) = 354. In the second column of the table, (d) and (r) stand for donor and recipient, respectively. HR, hazard ratio; CI, confidence interval. †Not applicable (N.A.) due to a low minor allele frequency. No SNP correlated with overall survival (statistical significance was determined using the Wald test).

Table S16. Multivariable regression of overall survival on donor and recipient SNPs

Gene	SNP (donor /recipient)	Additive model		Dominant model		Recessive model	
		HR (95% CI)	*p*	HR (95% CI)	*p*	HR (95% CI)	*p*
IL2	rs2069762 (d)	0.99 (0.84–1.16)	.879	0.96 (0.78–1.18)	.693	1.06 (0.75–1.48)	.753
IL6	rs1800796 (d)	0.90 (0.75–1.07)	.229	0.84 (0.68–1.05)	.119	N.A.†	N.A.
IFNG	rs2069765 (d)	1.09 (0.89–1.33)	.398	1.08 (0.86–1.35)	.522	N.A.†	N.A.
TGFB	rs1800469 (d)	1.05 (0.90–1.23)	.504	1.08 (0.84–1.39)	.539	1.06 (0.83–1.36)	.645
IL2	rs2069762 (r)	0.93 (0.79–1.09)	.351	0.88 (0.71–1.09)	.231	0.99 (0.70–1.38)	.932
IL6	rs1800796 (r)	1.02 (0.86–1.21)	.829	1.04 (0.84–1.29)	.725	N.A.†	N.A.
IFNG	rs2069765 (r)	0.89 (0.71–1.11)	.301	0.89 (0.69–1.13)	.330	N.A.†	N.A.
TGFB	rs1800469 (r)	1.00 (0.85–1.16)	.952	0.83 (0.66–1.05)	.125	1.21 (0.95–1.54)	.129

The results for each SNP were obtained by running separate Cox regressions under the three genetic models, indicated in the top row of the table, adjusted with HLA-C mismatch, ABO mismatch, donor CMV status, recipient performance status, disease stage, recipient age, donor age, and female donor–male recipient. Malignant disease patients without a previous transplantation history were analyzed (n = 822). The number of primary events (death) = 354. In the second column of the table, (d) and (r) stand for donor and recipient, respectively. HR, hazard ratio; CI, confidence interval. †Not applicable (N.A.) due to a low minor allele frequency. No SNP correlated with overall survival (statistical significance was determined using the Wald test).
